# Comprehensive Characterization of *Armoracia rusticana* Roots and Leaves: Physicochemical Properties, Functional Potential, and Nutritional Composition

**DOI:** 10.3390/ijms26199462

**Published:** 2025-09-27

**Authors:** Bianca Șuian, Sonia Amariei, Ancuța Petraru

**Affiliations:** Faculty of Food Engineering, Ștefan cel Mare University of Suceava, 720229 Suceava, Romania; bianca.suian@usv.ro (B.Ș.); ancuta.petraru@fia.usv.ro (A.P.)

**Keywords:** horseradish compounds, polyphenols, chlorophyll, carotenoids, vitamin C

## Abstract

The present study aimed to comprehensively characterize the physicochemical, nutritional, and functional properties of *Armoracia rusticana* leaves and roots, with a focus on their potential as sources of bioactive compounds. Quality parameters (color, moisture, titratable acidity, pH), macronutrient (proteins, fats, carbohydrates, fibers) and micronutrient (minerals, vitamins) content were determined. Polyphenolic profiles were evaluated using HPLC-DAD in two types of extracts: methanol–water (1:1, *v*/*v*) and deionized water. Flavonols (quercetin, kaempferol, myricetin), hydroxybenzoic acids (p-hydroxybenzoic, vanillic, caffeic), and hydroxycinnamic acids (chlorogenic, p-coumaric, rosmarinic) were identified. Freeze-drying proved effective in preserving thermolabile compounds, such as vitamin C (299.78 mg/100 g) and polyphenols (107.14 mg/100 g). Antioxidant capacity of the leaf extracts ranged between 74.52% and 76.90%, while pigment quantification revealed high levels of chlorophyll a (360.7 mg/100 g), chlorophyll b (110.03 mg/100 g), and total carotenoids (72.35 mg/100 g). FTIR spectroscopy was employed to assess molecular structures and functional group composition. Overall, the results support the valorization of *A. rusticana* leaves—an underutilized plant part—alongside roots, for applications in functional foods and nutraceutical development.

## 1. Introduction

Horseradish (*Armoracia rusticana*) is a perennial plant belonging to the botanical family *Brassicaceae* [1,2,3,4,5,6,7,8,9,10], also known as *Cruciferae* [11]. Along with horseradish, the family also includes other well-known plants such as broccoli, cabbage, cauliflower, arugula [1], turnip [12], and radish [13,14].

The origin of horseradish is believed to be in Eastern Europe, particularly countries such as Romania and Ukraine [4]. Horseradish has the advantage of being available throughout the entire year [3]. Compared to other species of the same family, horseradish is distinguished by a considerably higher content of glucosinolates [15].

Currently, research focuses mainly on horseradish roots, although the leaves are also considered to some extent, both due to the high content of glucosinolates [3,4] and isothiocyanates with anticancer properties [16]. Among the bioactive compounds found in horseradish, glucosinolates are the most abundant, with sinigrin being the predominant one [4,15,16]. These glucosinolate compounds are water-soluble and possess a repellent effect [4], and their levels vary depending on species, growth stage, and plant tissue such as root, leaf, seed, or stem [17]. Through various enzymatic, thermal or chemical degradation processes, their structure is modified, leading to the formation of isothiocyanates [16]. According to previous research, these compounds have a broad antibacterial potential [18], including multidrug-resistant clinical strains [16]. Horseradish-derived isothiocyanates exhibit antimicrobial effects against pathogens in the digestive tract [18] and oral cavity [19]. For example, Milan et al. [18] demonstrated that these compounds significantly inhibit the growth of pathogenic intestinal bacteria such as *Clostridium difficile* and *C. perfringens*, along with other microorganisms such as *Salmonella enterica*, *Aspergillus brasiliensis*, *Bacillus subtilis*, and *Proteus vulgaris*.

Of the total composition of isothiocyanates present in horseradish root, 78% are represented by allyl isothiocyanate [4], a compound responsible for the specific aroma [4,20]. Allyl isothiocyanate, obtained from horseradish by steam distillation, was incorporated into tofu, demonstrating its antimicrobial efficacy [20]. Horseradish distillates in the study conducted by Popovici et al. [16] demonstrated antimicrobial effects against the molds *P. notatum* and *Aspergillus niger* in food. At the same time, horseradish has also been shown to be effective against the bacterium *Listeria monocytogenes* [16,18]. Its presence represents a food safety issue since it has high resistance to freezing and drying temperatures, creating a biofilm even under extreme conditions [21].

The composition of horseradish has demonstrated a strong antimicrobial effect, managing to destroy bacteria such as *Escherichia coli* [4,18,19], *Salmonella typhimurium*, *Staphylococcus aureus* [4,18,19,20], and *Helicobacter pylori* [4,19], *Streptococcus mutans*, *Penicillium notatum*, *Bacillus cereus* [4,18], and *Vibrio parahaemolyticus*, and molds such as *Aspergillus flavus*, *Endomyces fibuliger*, *P. commune*, *P. corylophilum*, *P. dis color*, *P. palitans*, *P. polonicum*, *P. raqueforti*, *P. solitum*, and *Pichia anomala* [4].

In a previous study [22], the composition of lyophilized horseradish roots with potential antiallergenic effects was investigated, while the present study complements those findings by providing a comprehensive physicochemical, nutritional, and functional characterization of horseradish leaves and root.

## 2. Results

### 2.1. Determination of Powder Color

The values of the color parameters L*, a*, and b* are influenced by both the pigment composition of the raw material and its particle size [23]. In horseradish root, the main pigments are lutein and carotenoids [24], which are responsible for its distinctive shade. In leaves, chlorophyll a and b are the predominant pigments, giving them their characteristic green color [25].

During dehydration, color changes between the fresh and dried forms significantly affect the visual appearance of the finished product [13]. These changes, commonly observed in fruits and vegetables, are closely linked to browning reactions, whether enzymatic or non-enzymatic [13]. Green leafy vegetables are characterized by negative values of the a* parameter and high positive values of the b* parameter [26], a trend also confirmed by the results obtained in the present study. These changes, commonly observed in fruits and vegetables, are closely linked to browning reactions.

The overall color differences between the lyophilized samples and the white plate (ΔE_1_) and between the lyophilized and fresh samples (ΔE_2_) are presented in Table 1. Statistically significant differences were found for all color parameters (*p* < 0.0001).

Colorimetric determinations revealed significant differences among the samples, expressed as ΔE values. The color difference in horseradish root powder was 12.89, while in horseradish leaf powder it was 46.29. Comparison between fresh and dried roots showed a difference of 24.67, whereas the difference between fresh and dried leaves was 57.48, indicating a very pronounced color variation.

The color differences observed between dehydrated and fresh samples are a common phenomenon [13], associated with pigmentary and structural changes occurring during the freeze-drying process. In the case of leaves, which are rich in chlorophyll, the degradation of the green pigment, together with the exposure of carotenoids, led to a significant increase in the ΔE value. The ΔE_2_ value was found to be higher in horseradish leaf powder, indicating a greater total color difference between the dehydrated and fresh samples.

### 2.2. Moisture Analysis of Powders

Table 2 presents the results for the main quality parameters (moisture, water activity index, pH, acidity) and the main macronutrients (proteins, fats, carbohydrates) for powders obtained from horseradish root and leaves.

The high water content present in both fruits and vegetables classifies them as highly perishable products [13]. Although horseradish contains a lower water content (75%) compared to other root vegetables such as carrot, beetroot, celery, radish, or turnip, which have water contents ranging from 85.3% to 95.3% [3], it remains a product susceptible to degradation. Therefore, the application of effective preservation methods is essential to maintain its functional qualities and extend its shelf life.

Among the food preservation techniques, drying is among the most widespread and widely applied [13]. Although the drying process brings benefits, such as inhibiting enzymatic activity and limiting microbial growth, the technique used can affect the chemical composition and biological activity of plants [1,13]. Temperature is a key factor determining the quality of dehydrated products [13]. Due to their sensitivity to high temperatures, fruits and vegetables require fast and efficient drying processes [13]. While conventional drying affects the color, texture, but also the nutrients contained in the plants, freeze-drying confers quality to dried products, although the time and costs are higher [14].

Although the chemical composition of the powders is different, the oven drying process has the effect of uniformizing the residual water content. The moisture values determined for the lyophilized horseradish powder obtained from roots (7.13 ± 0.1%) and leaves (7.19 ± 0.12%) did not vary statistically significantly (*p* = 0.547), suggesting a relatively uniform composition in terms of moisture. Drying reduces free water and some of the bound water, and the temperature and duration applied remove water in a comparable way for both types of plant materials, with a moisture difference between the two samples of 0.84% compared to the minimum value.

### 2.3. Water Activity of Powders

In the dehydration process of fruits and vegetables, the main challenge is to reduce moisture to a level that inhibits microbial growth without compromising the sensory quality and nutritional value of the product [13]. In this context, determining water activity is essential, as it provides direct information regarding the microbiological stability of the dehydrated product and its ability to be safely stored. Water activity in foods directly influences both the rate of degradation reactions and the stability of products during storage [27].

The values obtained in the present study for the water activity in the lyophilized powder of horseradish root and leaves are 0.21 and 0.47, respectively, with a statistically significant difference between samples (*p* < 0.0001).

### 2.4. Titratable Acidity of Powders

The powder acidity determined in the lyophilized powder of horseradish root (7.2 ± 0.1 meq/100 g) and leaves (7.22 ± 0.01 meq/100 g) did not show statistically significant differences (*p* = 0.748).

### 2.5. pH of Powders

pH refers to the level of hydrogen ion activity in an aqueous solution [28].

The pH values for the powders obtained from horseradish leaves and roots were 5.98 ± 0.01 and 5.74 ± 0.01, respectively, indicating a statistically significant difference in acidity between the two types of plant material (*p* < 0.0001). Both samples fall within the weakly acidic range; however, the slightly higher pH of the horseradish leaves (closer to neutral) suggests lower acidity compared to the root.

### 2.6. Ash Content of the Powders

The ash content of horseradish powder was 5.07 ± 0.01% in the root and 9.94 ± 0.74% in the leaves, with the value in the leaves being almost twice as high. A statistically significant difference was observed between the two samples (*p* < 0.0001).

### 2.7. Protein Content of the Powders

Due to their complex functional role, proteins are key components in food formulation and processing, conferring properties such as emulsification, fat and water retention capacity, gelation and foaming [29], aspects that directly influence the texture, appearance and acceptability of the final product.

Determining the protein content is fundamental in the process of the valorization of vegetable raw materials, as it influences their applicability in various food formulations through the ability to retain fats and water, gelation and foaming [23].

Lyophilization, by reducing the moisture content of the powders to approximately 7%, leads to a significant increase in the concentration of proteins relative to the dry mass. For example, while fresh horseradish root contains 4.5% protein [3], in the lyophilized form, this value increased to 12.35 ± 0.43%. In contrast, horseradish leaves have been recognized as a richer source of proteins compared to the root, a fact also supported by a recent study by Justyna et al. [30], and this is reflected in our results as well—with a significantly higher protein content observed in the leaves (27.22 ± 0.59%, *p* < 0.0001).

### 2.8. Fat Content of the Powders

The total fat content in horseradish root powder is 1.07 ± 0.06%, while in the leaves it is over three times higher, namely 3.37 ± 0.14%, indicating a significant difference between the samples (*p* < 0.0001).

### 2.9. Fiber Content of the Powders

Regarding the fiber content for the two analyzed samples, significant differences were identified between them (*p* < 0.0001): the powder obtained from horseradish root contains 9.42 ± 0.01% fiber, while that obtained from horseradish leaves contains 9.72 ± 0.02% fiber.

### 2.10. Carbohydrate Content and Energy Value of the Powders

Horseradish root was noted for its high percentage of carbohydrates, namely 65.05 ± 0.02%, while horseradish leaves showed a slightly lower content, namely 42.83 ±1.35.

There were significant differences (*p* = 0.0007) between the two horseradish samples analyzed in terms of energy value: 319.22 ± 2.26 kcal/100 g root and 310.56 ± 5.92 kcal/100 g leaves. The higher fat and protein content in the leaves compensates for the higher carbohydrate content found in the root. The elevated energy values observed in both samples are primarily due to the freeze-drying process, which concentrates carbohydrates, proteins, and fats. For reference, the energy value of 100 g of fresh horseradish root is approximately 81 kcal, mainly due to its carbohydrate and protein content [3].

### 2.11. Mineral Elements in Powders and Soil

The profile of the ten mineral elements in the root and leaves of *Armoracia rusticana* and the soil in which they were grown are expressed in Table 3 in order to establish the relationship between soil availability and plant accumulation. Statistical analysis showed that all five common minerals—sodium, magnesium, chromium, zinc, and silver—had significantly different concentrations between the horseradish root powder and that from horseradish leaves (*p* < 0.05 for each mineral).

Following the determinations, a richer mineral composition was identified in horseradish roots (755.82 mg/100 g) compared to horseradish leaves (61.5 mg/100 g), an effect possibly due to the physiological role of the root in the absorption and accumulation of minerals from the soil, which recorded double mineral values (1447.37 mg/100 g). Horseradish leaves, although in lower concentrations, also included valuable minerals, such as sodium, magnesium, chromium, zinc, and silver.

Sodium and magnesium are part of the mineral composition of the analyzed samples, representing a rich source of electrolytes and contributing to the proper functioning of the human body [26]. In the roots, the most abundant mineral identified is calcium (530.41 ± 7.75 mg/100 g), corresponding to approximately two-thirds of the recommended daily intake of 800 mg/day for adults and children [26].

Although manganese was not identified in freeze-dried horseradish leaves or in soil, the value obtained for manganese in freeze-dried horseradish roots is almost identical to powders obtained from cabbage, broccoli and asparagus (0.2 mg/100 g) [31]. Cobalt and gallium are the only minerals that were not detected in either the leaves or the horseradish root, which suggests that the *Armoracia rusticana* species does not accumulate this mineral or that the concentration in the soil was too low (10.64 ± 0.05 mg/100 g) to be transferred to the plant.

### 2.12. Functional Properties of the Powders

**Figure 1:**
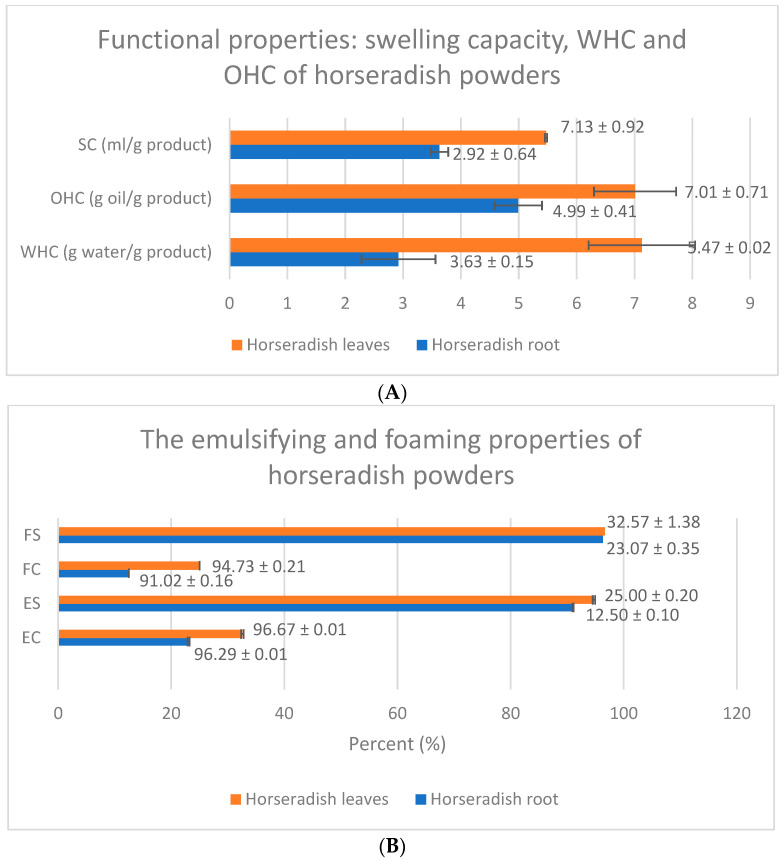
Functional properties of freeze-dried powders from horseradish root and leaves. (**A**) Hydration and fat interaction properties—SC = swelling capacity; OHC = oil-holding capacity; WHC = water-holding capacity; (**B**) Emulsifying and foaming properties—FS = foaming stability; FC = foaming capacity; ES = emulsion stability; EC = emulsion capacity.

Figure 1 shows the graphs for the functional properties of *Armoracia rusticana* powders.

#### 2.12.1. Water Holding Capacity

The presence of a large number of polar groups in the protein structure, capable of forming interactions with water molecules, may represent an important factor contributing to the high values of water retention capacity [26]. The importance of this characteristic is closely related to other functional properties [32].

In the context of water and oil retention capacity, proteins play an essential role, favoring the absorption of water on the surface. Horseradish leaf powder showed a higher water retention capacity (7.13 g ± 0.92 water/g product) compared to the powder obtained from horseradish roots (2.92 ± 0.64 g water/g product), the difference being statistically significant (*p* = 0.003). This difference can be correlated with the different protein content of the two powders.

#### 2.12.2. Oil Holding Capacity

Oil absorption capacity (OHC) plays a significant role, as fats help to retain aromas [33] and enhance the sensory experience [32]. High OHC reflects the potential of the ingredient to fix aromatic compounds and to actively participate in the formation and stabilization of the food matrix [26].

Powder obtained from horseradish leaves showed a higher oil retention capacity (7.01 ± 0.71 g oil/g product) compared to powder from horseradish roots (4.99 ± 0.41 g oil/g product), the difference being statistically significant (*p* = 0.013).

#### 2.12.3. Density

Density is an important property for packaging [32]. The powder obtained from horseradish roots showed a considerably higher density (0.32 g/cm^3^) compared to the powder from leaves (0.18 g/cm^3^), the difference being statistically significant (*p* = 0.016). The higher solids content of horseradish roots, together with the increased porosity of the leaves, determined by the presence of numerous intracellular spaces, may justify the almost twofold difference in density observed between the two types of powder.

#### 2.12.4. Swelling Capacity

The swelling capacity of powders is of great importance in the context of using the matrix of food or functional products and packaging, as it influences the texture and viscosity. The swelling capacity of the powder obtained from horseradish leaves (5.47 ± 0.02 mL/g) significantly exceeded (*p* < 0.0001) that of the powder from the root (3.63 ± 0.15 mL/g).

#### 2.12.5. Emulsifying Capacity and Emulsion Stability

Emulsifying properties are important characteristics through which proteins and other amphoteric molecules contribute to the development of foods [33]. These properties are usually evaluated by two main indicators: emulsifying capacity, which reflects the ability of proteins to generate an emulsion, and emulsion stability, which highlights their ability to maintain the emulsified system stable for a certain period of time [23,33]. In addition to proteins, carbohydrates such as starch and fiber can improve emulsion stability by acting as physical barriers between oil droplets and reducing the rate of coalescence [33].

The powder obtained from horseradish leaves had a significantly higher emulsification capacity (32.57 ± 1.38%) compared to the powder from the root (23.07 ± 0.35%), the difference being extremely statistically significant (*p* = 0.0003).

According to Figure 1, the height of the emulsion layer remained relatively constant at the analyzed time intervals (10, 20, and 30 min), more constant for both powders (91.02 ± 0.16%, respectively, 94.73 ± 0.21%).

#### 2.12.6. Foaming Properties and Foam Stability

Foaming properties, such as foaming capacity and foam stability, play an important role in achieving the aerated texture of food products, as they depend on the ability of proteins to efficiently distribute at the air–liquid interface. Since this characteristic is largely influenced by protein content, horseradish leaf powder recorded a double value (25 ± 0.2%) compared to root powder (12.5 ± 0.1%), the difference being highly statistically significant (*p* < 0.0001). Foam stability is also higher (96.66%) compared to roots (96.29%). Foaming capacity is essential for gas retention in fortified foods, such as bread and biscuits [23]. Additionally, proteins, carbohydrates [33] and amino acids contribute to foam formation, thereby enhancing the applicability of these raw materials in products such as toppings and beverages [32].

#### 2.12.7. Gelling Properties

To ensure the retention of water, sugars, flavors, and other ingredients in food products, the formation of a stable gelatinous network is essential [23]. The concentration and type of proteins and carbohydrates,, along with the presence of minerals and fibers, contribute to the development of a three-dimensional network that remains stable under pressure [32]. Additionally, pH and other physicochemical parameters have a significant impact on the gelation process [32]. In this study, horseradish leaf powder demonstrated the ability to form a gel at concentrations of 10% and 15%, whereas horseradish root powder exhibited this property only at a 15% concentration. Neither powder was able to form a gel at a 5% concentration.

### 2.13. Fourier Transform Infrared (FTIR) Spectra

**Figure 2:**
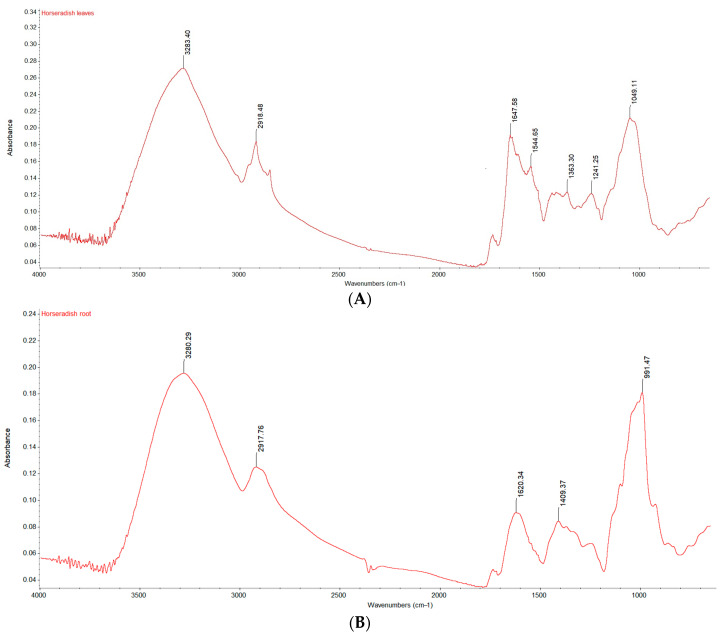
Infrared spectra obtained by the FTIR method for lyophilized powders: (**A**) horseradish leaf powder; (**B**) horseradish root powder.

Fourier Transform Infrared-Attenuated Total Reflectance (FTIR-ATR) is a green, fast, and innovative technique used to identify chemical or biochemical substances by detecting their molecular vibrations, such as torsion, rocking, stretching, bending, and scissoring [34,35]. Figure 2 illustrates the graphical representations of the infrared spectra obtained by the FTIR method for the powder obtained from horseradish leaves (A) and the powder obtained from horseradish root (B).

The FT-IR spectra showed seven wave numbers in three different spectral regions, namely single bond (4000–2500 cm^−1^), double bond (2000–1500 cm^−1^), and fingerprint (1500–600 cm^−1^) [36]. The samples show similar band positions, but the intensity varies based on the amount and type of functional groups present. Moreover, in leaves, three additional absorption bands were found.

In the first region, the absorption wavenumbers at 3280.29–3283.40 cm^−1^ can possibly be attributed to the hydroxyl stretching vibrations [37]. The spectral bands at 2917.76–2918.48 cm^−1^ were possibly attributed to asymmetric methylene stretching, mainly associated with the hydrocarbon chain found in fatty acids [38,39].

Two peaks in the double bond region are characteristics of amide I and II stretching, namely 1620.34–1647.58 due to C=O stretching vibrations and 1544.65 due to N-H bending [40,41].

The band 1241.25 cm^−1^ is specific to carbohydrates (cellulose, hemicellulose, lignin, and pectin) and consists of C-OH vibrations [42]. A weak band in the horseradish leaves was detected at 1363.30 cm^−1^ due to the deformation of the methyl group [43]. The spectral band at 991.47–1049.11 cm^−1^ is characteristic of glucosinolates, key compounds in horseradish [44].

### 2.14. Antioxidant Activity by DPPH Assay

Antioxidants play a key role in maintaining health [45], in mitigating the toxic effects associated with various conditions [32]. In this study, the antioxidant capacity of extracts from freeze-dried horseradish leaves was assessed by their ability to scavenge the DPPH radical. The results showed a high antioxidant activity for the extracts, with a slightly higher value for the deionized water extract (76.90 ± 1.2%) compared to the methanol–water (1:1, *v*/*v*) extract (74.53 ± 0.92%). Statistical analysis yielded a *p*-value of 0.1572, indicating no significant difference between the two extracts at the 0.05 significance level.

### 2.15. Chlorophyll and Carotenoid Content

Chlorophyll and carotenoids are leaf pigments, and any changes that occur between them indicate the effect of stress felt by plants as a result of environmental factors [46].

Chlorophyll is the photosynthetic green pigment [47] in the chloroplast of plants, and the ratio between chlorophyll a and chlorophyll b varies between 2.5 and 3.5, depending on the light specific to the environmental conditions [46]. This range was also confirmed in the case of the analysis of the powder obtained from lyophilized horseradish leaves, where the a/b ratio was 3.27. According to the specialized literature, a ratio value between 3.0 and 3.8 suggests that the leaves developed under full sunlight, while a value between 2.5 and 3.5 is indicative of full leaf maturation [48]. This ratio can also represent a biochemical marker of pollution, when the value is reduced [46]. Less commonly, the ratio between chlorophyll and carotenoids represents a sensitive indicator for differentiating between natural long-term senescence and senescence caused by environmental stress [46]. In the case of lyophilized horseradish powder this ratio was 6.50.

In plants, carotenoids contribute to photosynthesis by transferring light captured by chlorophyll a, and in the body they contribute to the synthesis of vitamin A [47]. The total value of carotenoids identified in horseradish leaves was 72.35 mg/100 g.

Chlorophyll a was present in the highest amount, with a value of 360.7 ± 0.57 mg/100 g dry matter, followed by chlorophyll b, with a value of 110.03 ± 1.23 mg/100 g dry matter. Chlorophyll c was not detectable under the conditions of the applied method, as it is mainly specific to algae [25]. In total, the lyophilized horseradish leaf powder contained 470.73 ± 3.39 mg/100 g dry matter of the powder.

### 2.16. Vitamin C Content

The product matrix, defined by its physicochemical composition (such as water, fiber, lipid or protein content), can significantly influence the stability of bioactive compounds. Depending on the interactions between these compounds and the matrix components, the effects of low temperatures can vary from one vegetable to another [2]. In the case of horseradish roots, a protective effect of the active compounds was recorded during the freeze-drying process [2,14]. Also, in the case of horseradish and lovage leaves, it was determined that the freeze-drying process preserves the highest proportion of the total polyphenol content and the flavonoid content [1]. In the case of several root vegetables studied by Marik [14], the freeze-drying process determined the lowest losses of vitamin C (celery, carrot, fennel, purple carrot, parsley, and yellow carrot).

Due to its sensitivity to temperature, vitamin C is frequently used as a quality indicator in the thermal processing of food products [49]. Horseradish contains a generous amount of vitamin C, three times higher than in citrus fruits [22], and is known as a remedy used against scurvy [10]. Vitamin C plays a role in the regeneration of quercetin, prolonging its biological activity through the complementarity of antiviral and immunomodulatory effects [50].

**Figure 3:**
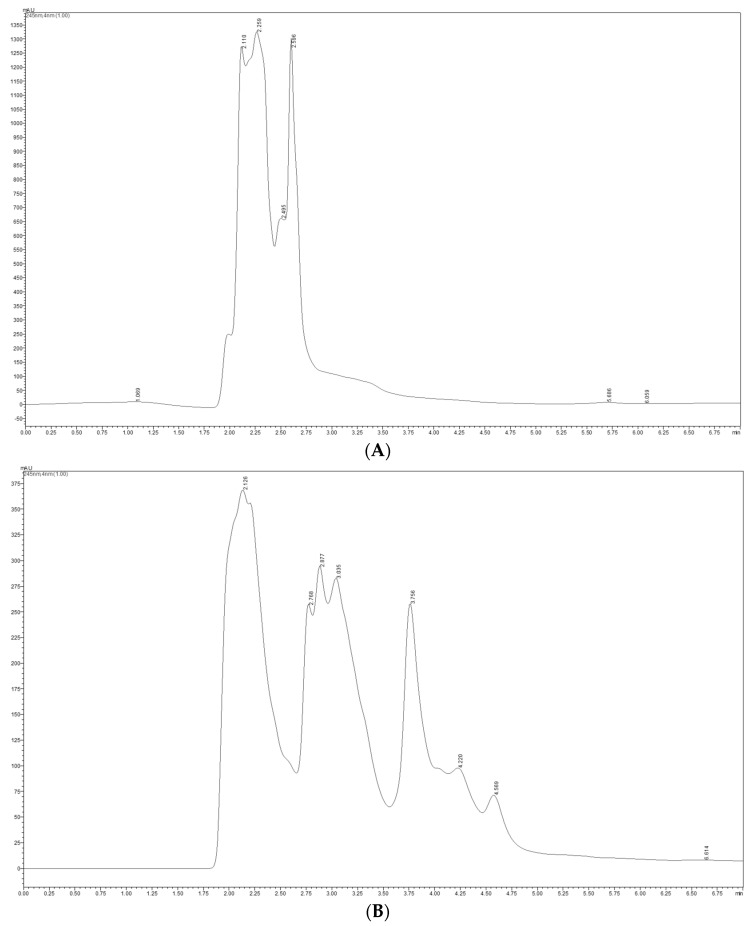
Chromatograms of vitamin C in freeze-dried horseradish leaves in methanol–water (1:1, *v*/*v*) extract (**A**) and aqueous extract (**B**).

Figure 3 shows the chromatograms obtained by HPLC analysis of vitamin C from lyophilized horseradish leaves, for the extract made with methanol–water (1:1, *v*/*v*) (A), respectively, for the extract with deionized water (B).

For 100 g of lyophilized horseradish leaf powder, a vitamin C value of 299.78 ± 2.89 mg/100 g was obtained for the methanol–water (1:1, *v*/*v*) extract and a value of 115.21 ± 0.73 mg/100 g for the aqueous extract. Statistical analysis indicated a *p*-value = 0.0001, which shows a highly statistically significant difference in the case of vitamin C (*p* < 0.001).

### 2.17. Phenolic Compound Profile

Flavonoids are substances with higher antioxidant potential than α-tocopherol, found in most parts of plants, but especially in leaves, roots, and bark [7]. They are attributed important properties, such as antimicrobial, anticancer, anti-inflammatory, antioxidant [7,44], antiallergenic [22], and antiviral [7]. Flavonoids also possess neuroprotective properties relevant for neurodegenerative diseases, including Alzheimer’s disease [44].

Quercetin has a strong antiviral effect demonstrated in the inhibition of herpes virus, cytomegalovirus, varicella-zoster virus [51], but also influenza [52], including coronaviruses [53]. In addition to its antiviral activity, quercetin also exhibits broad-spectrum antimicrobial properties, acting against various Gram-negative and Gram-positive bacteria [30].

Another category of phytochemicals is represented by phenolic acids, with important health roles and potential antioxidant, anticancer, neuroprotective, anti-inflammatory, and antimicrobial benefits [52].

Phenolic compounds are known for their strong antioxidant activity, and their efficiency depends on the way the molecular conformation is structured [45]. At the same time, the variety, climate, storage, and processing methods are the main factors that influence the composition of plants in phenolic substances [5]. The anatomical area of the plant, the solvent, and the processing method also play significant roles in the identification of bioactive compounds [44].

While most existing studies focus on horseradish roots, research on leaves remains limited [44]. Nevertheless, horseradish leaves are known to contain high levels of phenolic compounds and antioxidants [45].

**Figure 4:**
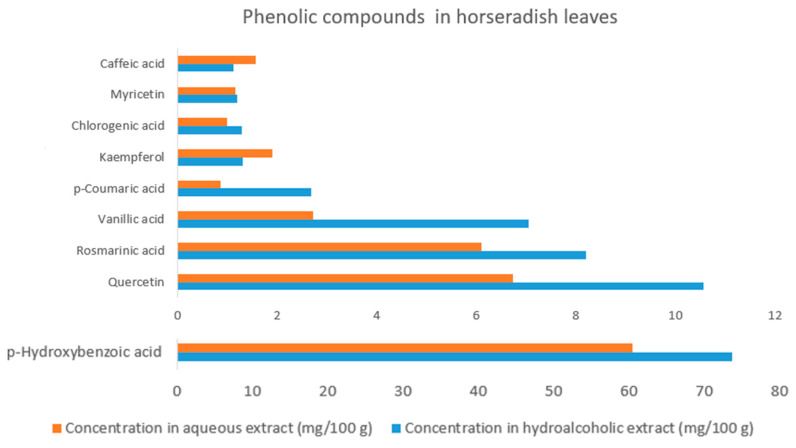
Content of identified phenolic compounds in horseradish (*Armoracia rusticana*) leaves for two different solvents (deionized water and methanol–water 1:1, *v*/*v*).

A total of nine phenolic compounds were identified in the freeze-dried horseradish leaves studied by HPLC analysis (Figure 4). Comparative analysis of polyphenols by solvent showed that most compounds showed significant differences (*p* < 0.05). The exception was rosmarinic acid and myricetin, for which the p values were higher (*p* = 0.055 and *p* = 0.306, respectively), indicating that the variation between solvents was not statistically significant for these two polyphenolic compounds.

Leaves harvested in June showed high concentrations of phenolic compounds, as expected from the literature, which indicates this period as optimal for the accumulation of these secondary metabolites and antioxidant substances [45].

In both types of solvents analyzed, *p*-hydroxybenzoic acid was found in the highest proportion, with a value of 73.69 ± 1.15 mg/100 g in the methanol–water (1:1, *v*/*v*) extract and 60.48 ± 0.1 mg/g in the aqueous one, while quercetin is in second place with a value of 10.56 ± 0.22 mg/100 g in the methanol–water (1:1, *v*/*v*) extract and 6.75 ± 0.05 mg/g in the aqueous one. Together, the two compounds account for 78.64% in the methanol–water (1:1, *v*/*v*) and 81.42% in the deionized water extract.

These values were followed by rosmarinic acid, with 8.21 ± 0.72 mg/100 g for the methanol–water (1:1, *v*/*v*) extract and 6.11 ± 0.03 mg/100 g for the deionized water extract, along with vanillic acid, with 7.06 ± 0.03 mg/100 g in the methanol–water (1:1, *v*/*v*) extract and 2.74 ± 0.04 mg/100 g in the aqueous extract. In the methanol–water (1:1, *v*/*v*) extracts, the remaining acids were identified in the following order: *p*-coumaric acid (2.7 ± 0.14 mg/100 g), kaempferol (1.31 ± 0 mg/100 g), chlorogenic acid (1.3 ± 0.03 mg/100 g), myricetin (1.3 ± 0.12 mg/100 g), and caffeic acid (1.13 ± 0.02 mg/100 g). The order of the other five phenolic compounds identified in the aqueous extracts was as follows: kaempferol (1.91 ± 0.02 mg/100 g), caffeic acid (1.58 ± 0.06 mg/100 g), myricetin (1.16 ± 0.07 mg/100 g), chlorogenic acid (1 ± 0.01 mg/100 g), and *p*-coumaric acid (0.86 ± 0.03 mg/100 g).

Among the nine identified phenolic compounds, seven showed higher solubility in the methanol–water (1:1, *v*/*v*) extract, except for kaempferol and caffeic acid, which were more soluble in the deionized water extract, exhibiting higher values.

## 3. Discussions

### 3.1. Physicochemical Parameters

For the L*, a*, and b* coordinates of horseradish leaves, values close to those specific to fresh radish leaves (L* = 46.47, a* = −11.45, b* = 28.64) and lettuce leaves (L* = 67.04, a* = −14.74, b* = 44.96) were recorded [26].

Similar chromatic parameters (L* = 49.70 ± 0.28, a* = −5.80 ± 0.12, b* = 11.70 ± 0.12) were also identified in the study conducted by Kaur and Bhatia for radish leaf protein concentrates [23].

When comparing the brightness (L*) values of freeze-dried horseradish root with those reported in a study using heat-drying methods, significant differences are observed. These differences are attributed to the distinct effects of the two dehydration methods on the structure and pigments of the plant material. In the heat-dried samples, the L* values were 51.07 at 50 °C, 48.95 at 70 °C, and 38.96 at 80 °C [13]. It is also noteworthy that increasing the drying temperature leads to a decrease in lightness (L*) values.

This phenomenon is attributed to the partial degradation of phenolic compounds and the formation of brown pigments, which results in a darker color and consequently lower L* value. In contrast, freeze-drying, by avoiding exposure to high temperatures, better preserves the original pigment structure and composition, yielding lightness values closer to those of the fresh material.

Compared to the data reported by Maric et al. in the literature for other root vegetables, the obtained values for brightness were found to be similar to those recorded for horseradish root, in the case of celery powders (L* = 85.22), fennel (L* = 80.81), parsley (L* = 81.62), yellow carrot (L* = 81.44), and similar to horseradish leaves, in the case of purple carrot (L* = 54.53) [14]. The same study indicated comparable values of the a* parameter for freeze-dried carrot (a* = 26.65) and fresh yellow carrot (a* = 24.64), similar to those recorded for freeze-dried horseradish leaves and an almost identical value of freeze-dried parsley (a* = 13.17) with freeze-dried horseradish root powder.

In the study conducted by Tomsone et al. on horseradish root and leaf juice, freeze-dried samples with 93% dry matter were obtained, corresponding to a moisture content of 7%, which is comparable to the results of the present study [8]. Similarly, radish roots, a plant related to horseradish, have a moisture content of 95.24 ± 0.29%, which is close to the present findings [26].

Water activity values obtained for horseradish root powder are comparable to those reported by Guragain and Mukesh for horseradish root dried at 50 °C with a blanching pretreatment. The water activity values obtained for horseradish root powder are comparable to those reported by Guragain and Mukesh for horseradish root dried at 50 °C with a blanching pretreatment [13].

When comparing the pH value of freeze-dried horseradish root with that of several genotypes of fresh turnip root, a plant related to horseradish, as studied by Sengul et al., a noticeable difference is observed due to the varying water content of the samples [31]. In the case of fresh turnip root, acidic and basic compounds are more diluted due to the high moisture content, resulting in a pH closer to neutral. In contrast, freeze-drying removes almost all water, and reconstituting the sample for measurement produces a solution with a relatively higher concentration of ionizable compounds, which can lead to a lower pH. Thus, the obtained values reflect not only the intrinsic chemical composition of the material but also its physical state and degree of hydration.

### 3.2. Functional Properties

The values obtained for the water retention capacity were superior for both types of powders studied, compared to those from horseradish root subjected to different thermal treatments (1.49–1.98 g/g). The results obtained highlight the potential of these powders, especially that from lyophilized horseradish leaves, to be used in aqueous food formulations as functional ingredients [23], as well as in pastry and bakery products, due to their ability to improve the texture of doughs by assimilating and retaining water [32].

In a study conducted by Guragain and Mukesh, which examined the effect of different drying treatments (drying at 50 °C, 70 °C, 85 °C, blanching fallowed by drying) on horseradish root, the density of the dehydrated samples was significantly higher, ranging from 0.69to 0.91 g/cm^3^ [13]. Also, blanching pretreatment and drying temperature were shown to have a significant influence on the density of dehydrated horseradish root [13].

A similar emulsification capacity was reported in the literature for radish leaf protein concentrate (48.1%) [29]. However, the emulsion stability of the powders analyzed in this study was higher than that of the radish leaf protein concentrate (47.8%) [23], indicating good emulsifying properties and satisfactory stability for both powders.

The foaming capacity of horseradish leaves, influenced by the percentage of protein they contain, presented values similar to those of protein concentrates obtained from radish leaves (20.4%) [23].

### 3.3. Nutritional Composition

Radish, a plant related to horseradish, shows the same pattern identified in the present study for ash content, with the root recording lower values (0.77 mg/100 g) compared to the leaves (1.70 mg/100 g) [26].

The protein content obtained for lyophilized horseradish root in this study is relatively close to the average value reported by Sengul et al. for several turnip genotypes (14.65%) [31], which may be attributed to the botanical relatedness of these root crops.

The calcium values obtained in the studied powders are comparable to those in radish leaf protein concentrates (623.22 ± 10.67 mg/100 g) [23]. The magnesium concentration in horseradish root is comparable to that in radish root (14.98 ± 0.02 mg/100 g), whereas manganese (0.07 ± 0.01 mg/100 g) and zinc (0.24 ± 0 mg/100 g) showed lower values compared to horseradish [26].

In fresh horseradish roots, zinc has a value of 1.4 mg/100 g, almost 30 times lower than in the lyophilized sample, and calcium has 78 mg/100 g, a value almost 7 times lower, due to the concentration of mineral substances through the lyophilization process [3].

### 3.4. Antioxidant Activity and Bioactive Content

Compared to the methanol–water (1:1, *v*/*v*) (96.93%) and aqueous (89.34%) extracts of horseradish roots [22], the leaves show a lower antioxidant capacity. In the case of leaves, the antioxidant capacity was higher in the aqueous extract than in methanol–water (1:1, *v*/*v*), the difference being 2.37%. The behavior was not similar in the case of horseradish roots, where the values were higher for the methanol–water (1:1, *v*/*v*) extract, with a difference of 7.59%.

The observed reverse trend can be attributed by the differing solubility of antioxidant compounds: in the leaves, water-soluble substances predominate, whereas in the roots, a significant proportion of the bioactive compounds are more soluble in alcoholic medium.

A similar relationship between root and leaf antioxidant activity was reported in a previous study evaluating the retention of bioactive compounds in freeze-dried horseradish juice, where the root showed higher antioxidant activity (78.90%) compared to the leaves (49.28%) [8]. These findings are in line with the current results, confirming a higher antioxidant capacity and density in the anatomical area of the roots, which typically accumulates greater amounts of glucosinolates and isothiocyanates.

When comparing the chlorophyll values obtained in the present study with those reported by Goyeneche et al., a significantly higher total chlorophyll content can be observed in freeze-dried horseradish leaves compared to fresh radish leaves—52.33 mg/100 g for leaves grown outdoors and 25 mg/100 g for those grown in a greenhouse [26].

The analysis of material obtained by pressing horseradish leaves in the study conducted by Tomsone et al. revealed low chlorophyll values, which decreased almost progressively with increasing drying temperature, a factor significant for all detected pigments [54]. Chlorophyll in the fresh material showed the highest values (chlorophyll a—244 mg/100 g, chlorophyll b—91 mg/100 g, total chlorophyll—335 mg/100 g), all lower compared to the results of the present study on freeze-dried leaf powder [54]. In the freeze-dried leaf pomace, the values were also lower than in the freeze-dried powder (chlorophyll a—145 mg/100 g, chlorophyll b—78 mg/100 g, total chlorophyll—223 mg/100 g), but higher than the values obtained after microwave and conventional drying at 40 °C, 60 °C, and 80 °C, which ranged between 104 and 133 mg/100 g for chlorophyll a, 23–69 mg/100 g for chlorophyll b, and 127–180 mg/100 g for total chlorophyll [54]. In the same study, the total carotenoid content in *Armoracia rusticana* leaves varied between 10 and 53 mg/100 g, depending on the thermal treatment applied [55]. Carotenoids exhibited an opposite trend, increasing with higher applied temperatures—a phenomenon also observed in chia seeds, where thermal treatment favored their extraction. A possible explanation is the inactivation of the enzyme responsible for their oxidation, resulting in values that increased proportionally with temperature [55]. Hower, the values obtained in the present study exceed those in the previously reported range, which may be attributed to differences in plant material (fresh leaves versus pomace) and processing methods, both of which significantly influence the variation in results.

Regarding vitamin C, the extraction efficiency was found to be higher in the methanol–water (1:1, *v*/*v*) extract due to the deeper penetration of the solvent into the plant matrix, contributing to a better release of the vitamin. A similar trend was identified for lyophilized horseradish root, where its value was 105.32 mg/100 g in the methanol–water (1:1, *v*/*v*) and 90.35 mg/100 g in the deionized water extract [22].

The results indicate that the freeze-drying process most effectively preserves vitamin C in horseradish leaf powder. A study conducted on horseradish leaf pomace showed that dehydration by freeze-drying retained the highest proportion of vitamin C (3.6 mg/100 g), compared to microwave drying (0.6 mg/100 g) or conventional drying at 40 °C, 60 °C and 80 °C, where it was not identified at all [54]. The vitamin C content is significantly lower compared to the values obtained in the present study, which can be explained by its affinity for the aqueous environment of the juice resulting from pressing, while in the pomace it is found only in small quantities, not being retained by the fiber matrix.

Variations in vitamin C concentration are common due to differences in geographical coordinates, harvest time, processing method, and extraction methodology [22].

The study conducted by Tomsone and Kruma concluded that the most suitable drying method is lyophilization, which most effectively preserves the polyphenolic compounds and antioxidant substances in horseradish and lovage leaves [1].

A similar study conducted by Tomsone et al. identified 14 phenolic compounds in the by-product obtained from pressed horseradish leaves using the HRLP technique [54]. The study demonstrated the effectiveness of freeze-drying in preserving phenolic compounds, outperforming fresh material, as well as samples dried by microwave-vacuum and hot air convection at 40 °C, 60 °C, and 80 °C [54]. Among all the methods compared, freeze-drying yielded the highest concentrations, even exceeding those found in fresh samples. Three phenolic compounds—4-hydroxybenzoic acid, chlorogenic acid, and p-coumaric acid—were consistently identified in both the current study and the investigation by Tomsone et al. [36]. The concentrations reported in the by-product (pomace) were considerably lower than those found in the present study. This discrepancy can be attributed primarily to differences in plant material, as whole leaves were analyzed in the current work, while the comparative study focused on pressed residues. Such variations highlight the significant impact of raw material composition on the recovery of phenolic compounds. Interestingly, chlorogenic acid showed similar levels across both studies, suggesting its relative stability regardless of plant matrix or processing conditions.

The presence of chlorogenic acid, *p*-coumaric acid, and caffeic acid was also confirmed in another study conducted by Tomsone and Kruma on freeze-dried horseradish leaves, where additionally rutin, catechin, sinapic acid, and ferulic acid were identified [1]. Among the common hydroxycinnamic acids identified, chlorogenic acid exhibited a higher value (2.15 mg/100 g), *p*-coumaric acid a lower value (0.17 mg/100 g), and caffeic acid an intermediate value compared to those obtained in the present study (0.93 mg/100 g) [2]. The reported value for caffeic acid in fresh leaves (1.22 mg/100 g) is similar and falls between the values obtained for the freeze-dried powder (1.13 mg/100 g in the methanol–water (1:1, *v*/*v*) extract and 1.57 mg/100 g in the aqueous extract). Similarly, the value for *p*-coumaric acid (1.14 mg/100 g) represents an intermediate value compared to those determined in the freeze-dried powder (2.7 mg/100 g in the methanol–water (1:1, *v*/*v*) extract and 0.86 mg/100 g in the aqueous extract). For all conventionally dried samples, the phenolic compound values showed lower contents of these acids. The use of different solvents, affecting the efficiency of phenolic compound extraction, may explain the observed differences [26].

For broader context, the values obtained in this study can be compared with those reported for other vegetables belonging to the same botanical family. In a study by Fusari et al., which investigated a range of fresh Brassicaceae vegetable, using deionized water and ultrasound-assisted extraction for phytochemical recovery, comparable values were reported [11]. In Brussels sprouts and white cabbage, identical caffeic acid values (1.3 mg/100 g) were identified, very close to the value of 1.3 mg/100 g obtained in the present study for the methanol–water (1:1, *v*/*v*) extract and 1 mg/100 g for the aqueous extract of horseradish leaves. Similar results, slightly higher than those for the methanol–water (1:1, *v*/*v*) extract, were recorded for *p*-coumaric acid in Brussels sprouts (3.4 mg/100 g), while a lower value was observed for green mustard (0.32 mg/100 g).

Therefore, depending on the plant matrix, the choice of solvent and extraction technique represent key factors for determining compounds with antioxidant properties [7].

Functional foods enriched with natural bioactive compounds are receiving growing attention for their ability to improve product functionality and stability [56]. In this context, the nutritional and technological properties of horseradish powders—such as high protein content, favorable hydration capacity, a rich mineral profile, and the presence of polyphenols and vitamin C—highlight their potential for application not only in food formulations, but also in sustainable packaging systems.

The advantages of freeze-drying in preserving sensitive compounds and ensuring long-term stability support the use of lyophilized horseradish as a value-added ingredient. Its incorporation into packaging matrices for products like cheese slices or processed meats may enhance both shelf-life and sensory attributes, aligning with current sustainability trends in the food industry.

## 4. Materials and Methods

### 4.1. Materials and Chemicals

Samples were collected from the same location, situated in the northeastern region of Romania, Bucovina. Roots were harvested in November 2024 at optimal maturity, while leaves were collected in June 2025. After cleaning, both roots and horseradish leaves were finely chopped using the Thermomix TM6 robot and immediately stored at −18 °C to minimize the degradation of heat-sensitive and oxidation-prone bioactive compounds, such as vitamin C and phenolics. Roots were peeled prior to chopping. Subsequently, the plant material lyophilized with the Biobase BK-FD12S freeze dryer at a pressure of 10 Pa for approximately 24 h, until the samples reached a temperature of −40 °C.

Following lyophilization, the dried roots and leaves were ground into a fine powder. The powders were then automatically sieved through a 200 μm mesh using a Retsch AS 200 Basic vibrator system (Retsch GmbH, Haan, Germany) equipped with a 200 μm sieve. The resulting powders were stored at −18 °C until further analysis.

All analytical grade reagents used were purchased from Sigma-Aldrich (Darmstadt, Germany). Stock solutions used for mineral standards for Na, Mg, Ca, and Mn were obtained from Fluka (Milan, Italy), while standards supplied by Cr, Co, Ga, Ag, and I were obtained from Merck (Darmstadt, Germany). pH meter calibration solutions were purchased from the manufacturer (Hach Company, Loveland, CO, USA).

### 4.2. Measurement of Color

The color of the powders obtained from horseradish roots and leaves was measured using a Konica Minolta CR-400 colorimeter (Konica Minolta, Tokyo, Japan) [57], after thoroughly homogenizing the powders. Color was determined using the CIELAB system (L*, a*, b*), where L* represents lightness, a* varies between green (−) and red (+), and b* between blue (−) and yellow (+) [13,26]. Before measurements, the colorimeter was calibrated using a standard white reference plate ($L_{0}$* = 94.17, $a_{0}$* = −0.57, $b_{0}$* = 4.14), included in the instrument kit.

The total color differences between freeze-dried powders and fresh horseradish were determined using Equation (1) [13]:

(1) $$∆E=\sqrt{{\left(L-L_{0}\right)}^{2}+{\left(a-a_{0}\right)}^{2}+{\left(b-b_{0}\right)}^{2}}$$

where

L, a, b = CIELAB color coordinates of the sample (measured values);
  L_0_, a_0_, b_0_ = CIELAB color coordinates of the reference (standard) sample

### 4.3. Determination of Moisture Content

Moisture content (U) was determined according to the official AOAC method 935.29 [58] by drying 5 g of sample in a ZRD A5055 oven (Dongguan Zhicheng Instrument Co., Ltd., Shanghai, China) at 105 °C until constant weight was achieved [59]. The moisture content was expressed as an average value calculated using the following calculation formula:

(2) $$U\left(\%\right)=\frac{m_{i}-m_{f}}{m_{i}}\times 100$$

where

$m_{i}$ = initial mass before drying;
  $m_{f}$ = final mass after drying.

### 4.4. Measurement of Water Activity

Water activity measurements were performed using an AquaLab 4TE device (Meter Group, Pullman, Washington, DC, USA).

### 4.5. Determination of Titratable Acidity

To determine titratable acidity, 5 g of powder were homogenized with 100 mL of distilled water for 20 min using a magnetic stirrer. The resulting solution was filtered through Whatman No. 9 filter paper to remove solid residues. Titratable acidity was determined by titrating 20 mL of the filtered solution in an Erlenmeyer flask with 0.1 N NaOH solution and 1% phenolphthalein as indicator. Three replicates were performed for each sample, and the final results were expressed in milliequivalents of NaOH required to neutralize the acidity, calculated according to Formula (3) [60]:

Titratable acidity (meq NaOH/100 g) = (V × N∕m) × 100(3)

where

V = volume of NaOH required for titration (mL);
  N = normality of the NaOH solution (eq/L);
  m = mass of the powder sample used in the analysis (g).

### 4.6. Determination of pH

The pH value was measured at room temperature using a portable digital pH meter HQ30d (Hach, Loveland, CO, USA). The pH meter was first calibrated with standard buffer solutions of pH 4.0 and 7.0, after which the glass electrode was immersed in suspensions with 1:10 dilution (powder–distilled water) [23,26].

### 4.7. Determination of Ash Content

Ash content was determined by incinerating the powders in a calcination oven at a temperature of 550 ± 10 °C for 4 h until complete mineralization of organic matter, according to International Standard ISO 2171 [61].

### 4.8. Determination of Protein Content

The crude protein content of the samples was calculated from the nitrogen content determined by the Kjeldahl method [31], which includes the digestion, distillation, and titration steps, using a conversion factor of 6.25 [29,30]. The following formula was used for the calculation:

(4) $$Proteins\left(\%\right)=\frac{\left(V_{sample}-V_{control}\right)\times z\times C\times f\times M_{N}}{m\times 1000}\times 0.14\times 6.25$$

where

V_sample_ = volume of HCl used for titration of the sample (mL);
  V_control_ = volume of HCl used for titration of the control (mL);
  z= stoichiometric factor (1 for HCl);
  C = concentration of HCl (mol/L);
  f = correction factor for the HCl solution (1);
  M_N_ = mass of nitrogen (14.007 g/mol);
  m = mass of the sample (g);
  1000 = conversion from mL to L.

### 4.9. Determination of Fat Content

To determine the fat content of the powder obtained from horseradish roots and leaves, a Soxhlet SER 148 extraction system (VELP Scientifica, Usmate Velate, Italy) was used with petroleum ether as the solvent. The fat content was calculated using the following equation:

(5) $$Fat\left(\%\right)=\frac{m_{2}-m_{1}}{m_{s}}\times 100$$

where

m_1_ = mass of the empty container (g);
  m_2_ = mass of the container with extracted fat (g);
  m_s_ = mass of the dried sample used for analysis (g).

### 4.10. Determination of Fiber Content

To evaluate the fiber content, the FibroTherm FT12 device (Gerhardt GmbH & Co. KG, Königswinter, Germany) was used in combination with the Megazyme K-TDFR-200A kit (Megazyme Ltd., County Wicklow, Ireland) [59] following the AOAC 985.29 method [62].

The total fiber content was determined using the following formula:

(6) $$Fiber\left(\%\right)=\frac{m_{2}-m_{3}-m_{4}}{m_{1}}\times 100$$

where

m_1_ = mass of the initial sample;
  m_2_ = mass of the bag with the residue remaining after digestion;
  m_3_ = mass of the empty bag;
  m_4_ = mass of the bag with the residue remaining after calcination.

### 4.11. Carbohydrate Content and Energy Value Determination

The carbohydrate content was calculated by difference, according to the following equation [25,31]:

(7) Carbohydrates (%) = 100 − (moisture + ash + proteins + fats + fiber)

The energy value was calculated using a formula adapted from Petraru and Amariei [59], with some modifications. Specifically, the fiber component (2 kcal/g) present in the original equation was excluded, in order to align with the conventional Atwater system, which does not include fiber as an energy-yielding nutrient. The final calculation used the standard conversion factors: 4 kcal/g for protein, 4 kcal/g for carbohydrates, and 9 kcal/g for fat:

(8) Energy value (kcal/100 g) = (4 × proteins) + (9 × fats) + (4 × carbohydrates)

### 4.12. Determination of Mineral Content

After calcination, the ash residue was treated with 730 µL of concentrated nitric acid (65%) to solubilize the mineral components [59]. The solution was then diluted to 50 mL with deionized water. Mineral content was determined using an ICP-MS 7500 spectrometer (Agilent Technologies, Santa Clara, CA, USA). A multielement stock solution (1000 mg/L for each element) was used to prepare the calibration standards, with a concentration of 1000 mg/L for each of the following elements, including Na, Mg, Ca, Cr, Mn, Co, Zn, Ga, Ag, and I [63]. The mineral composition of the powders was expressed as mg of mineral per 100 g of sample [23].

### 4.13. Determination of Functional Properties

#### 4.13.1. Water and Oil Holding Capacity

The water and oil retention capacity (WHC/OHC) were determined by adding 0.25 g of powder to 25 mL of distilled water and vegetable oil, respectively. The resulting suspensions were kept at room temperature for 1 h, then centrifuged at 4000 rpm for 25 min using an Ohaus Frontier 5718R centrifuge (Ohaus, Parsippany, NJ, USA) [24]. Subsequently, the supernatant was discarded, and the residue was weighed to calculate the retention capacity using the following formula:

(9) $$WHC/OHC\left(\frac{g_{water/oil}}{g_{product}}\right)=\;\frac{m_{2}-m_{1}}{m_{1}}$$

where

$m_{1}$= mass of dry residue (g);
  $m_{2}$ = mass of hydrated residue (g).

#### 4.13.2. Density

The density of the powder was determined by placing 5 g of sample into a 50 mL graduated cylinder ($V_{cylinder})$. Uniform rapeseed seeds were then added up to the 50 mL mark to fill the remaining volume [13]. The rapeseed was removed, and its volume was measured. The powder volume was calculated by subtracting the rapeseed volume ($V_{seed}$) from 50 mL. The bulk density was then determined using the following equation:

(10) $$Density\;\left(g/mL\right)=\frac{mass\;of\;the\;sample}{V_{cylinder}-V_{seed}}$$

#### 4.13.3. Swelling Capacity

In a centrifuge tube, 0.5 g of sample was mixed with 15 mL of distilled water. After homogenization, the tube was placed in a water bath (Memmert WNE 14, Memmert GmbH & Co. KG, Schwabach, Germany) and heated at 90 °C for 30 min. After cooling, the samples were centrifuged at 3000 rpm for 25 min (Ohaus Frontier 5718R, Parsippany, NJ, USA). The supernatant was then discarded, and the swollen pellet was weighed. The swelling capacity was determined using the following formula:

(11) $$SC\left(mL/g\right)=\frac{swollen\;gel}{m_{sample}}$$

#### 4.13.4. Emulsifying Capacity and Emulsion Stability

A 0.5% suspension was initially prepared, from which 30 mL was mixed with 10 mL of corn oil. The resulting emulsion was homogenized and immediately transferred into a 50 mL graduated cylinder, where the initial height of the emulsion layer was recorded. To assess emulsion stability, the cylinder was placed in a water bath (Memmert WNE 14, Memmert GmbH & Co. KG, Germany) at 80 °C for 30 min [59]:

(12) $$EC\left(\%\right)=\frac{h_{emulsified\;phase}}{h_{suspension}}\times 100$$

After heating, the height of the emulsion layer was measured again to evaluate changes from the initial value, using the following formula:

(13) $$ES\left(\%\right)=\frac{h_{final\;suspension}}{h_{suspension}}\times 100$$

where

h_emusion phase_ = height of the emulsified oil layer;
  h_suspension_ = total height of the suspension;
  h_final suspension_ = total height of the suspension after heating.

#### 4.13.5. Foaming Properties and Foam Stability

Two grams of powdered sample were added to 50 mL of distilled water and vortexed for 3 min at 7000 rpm [32]. The contents were transferred to a 250 mL graduated cylinder. The initial foam volume was recorded, and the final volume was measured after 1 h. The foaming properties were calculated using the following formula [32]:

(14) $$FC\left(\%\right)=\frac{V_{2}-V_{1}}{V_{1}}\times 100$$

where

$V_{2}$ = volume of solution after shaking (mL);
  $V_{1}$ = initial volume of solution (mL).

The volume of the solution was measured at 10 min intervals to assess stability over time [59]. The results were expressed using the following formula:

(15) $$FS\left(\%\right)=\frac{Foam\;volume\;measured\;at\;a\;given\;time}{Initial\;volume}\times 100$$

#### 4.13.6. Gelling Properties

To assess gelation properties, suspensions of three different concentrations (5%, 10% and 15%), were prepared using deionized water [33]. After homogenization, the suspensions were boiled in a water bath (Memmert WNE 14, Memmert GmbH & Co. KG, Germany) for 1 h, then rapidly cooled under a cold water jet and refrigerated at 4 °C [25,34]. Subsequently, the tubes were inverted to observe gel formation. The complete absence of flow upon inversion was used as the criterion for confirming the formation of a stable gel [59].

### 4.14. Fourier Transform Infrared Spectroscopy (FTIR) Analysis

Using a Thermo Scientific Nicolet iS20 spectrophotometer (Thermo Scientific, Karlsruhe, Germany) equipped with an ATR module, FTIR spectra were obtained at a resolution of 8 cm^−1^ through 64 scans recorded between 650 and 4000 cm^−1^. Data processing and spectra acquisition were performed using OMNIC software (version 9, Thermo Scientific, Waltham, MA, USA) as an integral part of the instrument’s operating system.

### 4.15. DPPH Radical Scavenging Assay Analysis

The DPPH free radical scavenging capacity was determined spectrophotometrically using a UV-VIS-NIR spectrophotometer (Shimadzu 300, Tokyo, Japan), by measuring the absorption at 517 nm, according to the method described by Șuian and Amariei [22]. The calculation of the inhibition percentage was performed using the following formula:

(16) $$Inhibition\;percent\left(\%\right)=\frac{A_{control}-A_{sample}}{A_{control}}\times 100$$

where

A_control_ = absorbance of the blank sample;
  A_sample_ = absorbance of the sample with extract.

### 4.16. Determination of Chlorophyll Content (a, b, c, and Total) and Carotenoids

To determine the chlorophyll content of horseradish leaves, an extract was prepared from 1 g of lyophilized powder and 25 mL of acetone (90%). This was subjected to double filtration and made up to the mark in a 25 mL volumetric flask. The absorbance of the solution was measured at 630 nm, 645 nm, 663 nm, and 750 nm for the determination of chlorophyll a, b, c, and total chlorophyll [64]. For the determination of carotene, the absorbance of the solution was measured at a wavelength of 470 nm [44,45,46].

### 4.17. Determination of Vitamin C content

The vitamin C content in horseradish powder was analyzed by high-performance liquid chromatography (HPLC) using a Shimadzu system (Kyoto, Japan) equipped with a diode array detector (model SPD-M20A) and a Zorbax Extended C18 column (150 × 4.6 mm, 5 µm; Agilent Technologies, Santa Clara, CA, USA), according to the method described by Șuian and Amariei [22].

### 4.18. Phenolic Compound Profile Determination

The phenolic compound profile was determined using an HPLC system (Shimadzu, Kyoto, Japan) equipped with a diode array detector (SPD-M20A), following the method described by Șuian and Amariei [22].

### 4.19. Statistical Analysis

All experimental determinations were performed in triplicate, and the results are reported as mean values ± standard deviation. Data processing and statistical analysis were performed using XLSTAT (version 2016.02, trial version, Addinsoft, Paris, France). Differences between means were evaluated using Student’s t-test, with results considered significant at *p* < 0.05.

## 5. Conclusions

In the current context of food system diversification and the growing demand for clean-label products, there is increasing interest in identifying natural ingredients that can replace synthetic additives while offering additional functional benefits. In this regard, the present study focuses on the roots and leaves of *Armoracia rusticana* (horseradish), a plant whose less-explored anatomical parts may hold promising nutritional and technological potential.

The samples were comprehensively analyzed in terms of physicochemical characteristics, functional properties, macronutrient and micronutrient composition, pigment profile, and structural features. Identified bioactive compounds—such as phenolic acids, flavonols, and vitamin C—are discussed in relation to their known biological activities, as reported in the literature, to highlight future application potential.

Multiple biological functions can be attributed to horseradish leaves due to their rich composition of phenolic compounds, such as flavonols (quercetin, kaempferol, myricetin), phenolic acids (*p*-hydroxybenzoic, rosmarinic, vanillic, *p*-coumaric, chlorogenic), and caffeic acids. The data indicated that their levels are closely related to the solvent used for extraction. Except for caffeic acid and kaempferol, which demonstrated higher solubility in the aqueous extract, the other seven phenolic compounds showed superior solubilization capacity in the methanol–water (1:1, *v*/*v*) extract, with consequently higher concentrations.

The analyses revealed a very high content of vitamin C, namely 299.78 ± 2.89 mg/100 g in the methanol–water (1:1, *v*/*v*) extract of horseradish leaves. Through the protective action of vitamin C on quercetin and other flavonoids, horseradish stands out as a natural matrix with remarkable synergistic potential. Moreover, the presence of chlorophyll and carotenoids highlights the contribution of pigments to the nutritional profile of horseradish leaves.

The study results indicate that *Armoracia rusticana* leaves represent a valuable source of bioactive compounds, making them suitable for incorporation into functional foods. They also possess functional characteristics (high water and oil binding capacity) and physicochemical properties (high protein and mineral content) that recommend them for integration into food formulations.

## Figures and Tables

**Table 1:** Colorimetric data L*, a*, b*, and ΔE for lyophilized horseradish powder samples.

Sample	L*	a*	b*	$\Delta E_{1}$	$\Delta E_{2}$
Root powder	85.87 ± 0.06	0.13 ± 0.06	13.97 ± 0.04	12.89	24.67
Leaf powder	55.23 ± 0.15	−14.90 ± 0.09	24.67 ± 0.08	46.29	57.48

L* = degree of brightness; a* = red (+)/green (–); b* = yellow (+)/blue (–); Δ$E_{1}$ = color difference with respect to the white plate; Δ$E_{2}$ = color difference with respect to the fresh sample.

**Table 2:** Physico-chemical quality parameters for lyophilized horseradish root and leaves.

Evaluated Parameter	Root	Leaves
Moisture (%)	7.13 ± 0.10	7.19 ± 0.12
Water activity (aw)	0.21 ± 0	0.47 ± 0
Titratable acidity (meq/100 g)	7.20 ± 0.10	7.22 ± 0.01
pH	5.74 ± 0.01	5.98 ± 0.01
Ash content (%)	5.07 ± 0.01	9.94 ± 0.74
Protein content (%)	12.35 ± 0.43	27.22 ± 0.59
Fat content (%)	1.07 ± 0.06	3.37 ± 0.14
Fiber content (%)	9.42 ± 0.01	9.72 ± 0.02
Carbohydrate content (%)	65.05 ± 0.02	42.83 ± 1.35
Energy value (kcal/100 g)	319.22 ± 2.26	310.56 ± 5.92

**Table 3:** Mineral profile of *Armoracia rusticana* roots and leaves and their associated soil.

No.	Mineral Element (mg/100 g)	Soil	Horseradish—*Armoracia rusticana*		*p*-Value
			Root	Leaves	
1.	Sodium (Na)	15.18 ± 0.08	8.91 ± 0.28	6.53 ± 0.48	0.0264
2.	Magnesium (Mg)	30.33 ± 3.53	18.11 ± 0.1	21.71 ± 0.23	0.0001
3.	Calcium (Ca)	406.07 ± 0.98	530.41 ± 7.75	nd	*-*
4.	Chromium (Cr)	0.66 ± 0.01	0.95 ± 0.03	0.67 ± 0.06	0.0286
5.	Manganese (Mn)	nd	0.23 ± 0.01	nd	*-*
6.	Cobalt (Co)	10.65 ± 0.01	nd	nd	*-*
7.	Zinc (Zn)	781.95 ± 0.28	45.59 ± 0.77	1.17 ± 0	0.0002
8.	Gallium (Ga)	2.02 ± 0.13	nd	nd	*-*
9.	Silver (Ag)	109.75 ± 0.16	79.96 ± 0.42	51.43 ± 0.07	0.0001
10	Iodine (I)	90.82 ± 0.89	71.69 ± 0.14	nd	*-*
	TOTAL	1447.43	755.85	61.51	

nd = not detected.

## Data Availability

The data presented in this study are available on request from the corresponding author.
